# It’s all about the relationship: The caregiver experience of supporting a person with advanced cancer going through an LSD microdosing trial

**DOI:** 10.1017/S1478951526101977

**Published:** 2026-02-26

**Authors:** Fiona Cottam, Alesha Wells, Cerys Clayden, Lisa Reynolds

**Affiliations:** Department of Psychological Medicine, Faculty of Medical and Health Sciences, University of Auckland, Grafton, Auckland, New Zealand

**Keywords:** Cancer caregivers, LSD, microdosing, MCP, distress, caregiver-patient relationship, dyad

## Abstract

Psychedelic therapies are gaining attention as a novel therapy for existential distress in cancer patients. However, such treatments are often stigmatized and the views of support networks are unknown. Caregivers play an essential role in supporting the wellbeing and decision-making of people with cancer. In the context of a trial investigating psychedelic therapy for cancer patients, understanding the experience of the caregiver is important.

**Objectives:**

The objectives of this research were to investigate the hopes, beliefs, perceptions, and experience of caregivers for advanced cancer patients undergoing a trial investigating a psychedelic-assisted therapy.

**Methods:**

Semi-structured interviews asked 15 caregivers about their experience at baseline and 1 month after their close associate had completed treatment in a feasibility trial where participants were randomized to receive either lysergic acid diethylamide (LSD) microdoses or placebo alongside meaning-centered psychotherapy (MCP). Blinded to condition, reflexive thematic analysis was used to analyze interview transcripts.

**Results:**

This study demonstrates the importance of bidirectional influences between caregiver and patient; the experience of one influences the experience of both. Caregivers were generally supportive of their close associate participating in a psychedelic-assisted trial, although some admitted hesitancy in them taking part. Caregivers described a desire to make the most of now, referring to the role of LSD microdose-assisted MCP as a means of accessing hope, improving the dyad relationship, and reducing existential distress.

**Significance of Results:**

Participation in trials investigating psychedelic-assisted MCP may offer hope for patients and their caregivers. Given the bidirectional relationship in wellbeing between cancer dyads, caregivers should be included alongside patients in such trials.

## Introduction

Advanced cancer and its associated life changes impact not only the patient but also their informal caregivers. Such caregivers are typically family members or close associates who play an integral role in the care and wellbeing of the patient. The caregiver is important in their role supporting the patient and also in themselves as a human being. In the context of a trial involving a novel and often stigmatised drug (lysergic acid diethylamide – LSD), understanding the experience and perspectives of the caregiver is important.

The caregiver’s responsibilities are diverse and complex, varying amongst patient support needs (Romito et al. 2013). Caregivers are an integral socio-emotional support for the cancer patient, influencing their treatment decision-making and acceptance of care (Kent et al. 2016). Caregiver perspectives are often overlooked by healthcare systems, including the degree of essential support they provide (Wang et al. 2018; Chong et al. 2023). Over recent decades, it has been recognized that the views and experiences of caregivers have been historically disregarded, with literature now showing the importance of recognizing the caregiver perspective in influencing engagement of patient participation in treatment and trials (Thomas et al. 2021; Reblin et al. 2022). Garnering the caregiver’s perspective of treatment modalities is important as their input can influence patient decisions and experiences of taking part in trials.

The caregiver perspective and their influence on patient behavior are pivotal in the context of novel treatment modalities. Psychedelic-assisted treatments, such as LSD microdosing, are often stigmatized due to misconceptions and beliefs about the drug (McGruddy et al. 2024). Psychedelic drugs have deeply rooted societal stigma through association with counter-culture movements perpetuated by hostile mass media representations (Orsini 2017; Williams 2018) and reports of risk (Orsini 2017). Alongside historical ties with counter-culture, psychedelics are classified by law in many countries as substances of abuse with no medicinal value (Misuse of Drugs Act 1975). In the context of cancer, societal misconceptions about psychedelic-assisted treatment may contribute to opposition by patients and their caregivers (Viña 2024). Especially significant within clinical trials of psychedelic-assisted therapy, is the prevalence of caregivers’ perceptions of LSD as they shape patients’ decision-making and willingness, or unwillingness, to participate.

Although research about psychedelic-assisted psychotherapy is still in its preliminary stages, there is promising evidence for the efficacy of psychedelic medicines alongside psychotherapies for conditions that have ceased responding to treatment (Cavarra et al. 2022; Schipper et al. 2024). Cancer patients have reported sustained long-term effects from LSD-assisted therapy for anxiety (Holze et al. 2024). Furthermore, evidence that microdoses of LSD are largely safe and non-addictive (Murphy et al. 2023) might alleviate some of the anxiety potential patients and caregivers feel.

Meaning-centered psychotherapy (MCP) has proven effective in reducing psychological distress and improving quality of life, social relationships, self-efficacy, and levels of hope amongst people with cancer (Vos and Vitali 2018). Recent evidence suggests that combined with psychological intervention, psychedelic substances are viable pharmacotherapy contenders in making therapies such as MCP work more effectively (Kamenov et al. 2016; Siegel et al. 2021). The current research investigated the hopes, beliefs, perceptions, and experiences of caregivers to advanced cancer patients who were undergoing a randomized-controlled feasibility trial investigating microdoses of LSD alongside MCP. This research aimed to investigate the caregiver perspective of supporting a patient within an intervention trial.

## Methods

The current research was secondary to a larger study (Psychedelic-Assisted Meaning-Centered Psychotherapy; PAM trial) investigating the feasibility, acceptability, and safety of LSD microdosing alongside MCP in advanced stage cancer patients. Further information about the PAM trial is reported elsewhere (Wells et al. 2024). In brief, participants in the PAM trial received individual MCP weekly for 7 weeks (Breitbart and Poppito 2014), and were randomly assigned to receive either 13 microdoses of LSD, or the same number of placebo doses. The PAM study was double-blinded, so neither the participants, caregivers, researchers, or MCP therapists knew whether participants had received LSD or placebo.

### Participants

The purpose of the current work was to understand the caregiver’s experience of supporting a patient going through the PAM trial. Participants were caregivers nominated by the PAM trial participant as a person closely associated with them, upon whom they may rely for personal care, emotional support, and/or domestic arrangements in an informal (i.e., unpaid) role. Participants also needed to speak English fluently and be over the age of 18 years.

### Materials

Interviews were semi-structured with a set of questions that elicited information about the caregiver’s experiences and attitudes. The semi-structured nature of the interview meant that a conversational tone was established, encouraging the caregiver to talk freely about their experience. It allowed probing by the interviewer in order to collect greater detail about the effect of the trial on the caregiver and allow clarification or further exploration when needed.

Caregiver participants completed a demographic questionnaire prior to involvement in the interviews, collecting information on their age, ethnicity, and relationship to the patient participant.

### Procedure

Caregiver participants of the current work took part in two interviews: (1) a baseline interview timed alongside the PAM trial participant’s first treatment session, and (2) a follow-up interview, held approximately 1 month after the PAM participant’s final treatment. Interviews were conducted by the researcher F.C.

Interviews were transcribed verbatim using Microsoft Word and amended according to the notation system described in Braun and Clarke (2013). Reflexive thematic analysis was chosen due to its flexibility in epistemological approach and because it allowed details of the data to provide a rich interpretation of experience (Braun and Clarke 2006). The primary researcher (F.C.) identified preliminary codes and themes in interview transcripts, which were reviewed through an iterative process with L.R., A.W., and C.C. Through this iterative process, themes were adjusted to accurately reflect caregiver perspectives of supporting a patient taking part in the PAM trial. The topics that held most salience for participants (for more detail see Byrne 2022) signaled the themes underlying the experience of being a caregiver of a participant in the PAM trial. All researchers and participants were blinded to the condition to which PAM participants had been randomized throughout the process of interviewing, analysis, theme generation, and manuscript production; therefore, this study did not assess differences in caregiver perspective between placebo and LSD-microdosing patients in the PAM trial.

## Results

Each caregiver participant in this study corresponded to 1 of 15 unique cancer patients participating in the PAM trial. They were mostly spouses or long-term partners of cancer patients who had participated in the PAM trial (*n* = 13), as well as one who was a parent, and one who was a daughter. The sample included a wide range of ages from mid-30s to mid-80s and most participants were NZ European (*n* = 12), and female (*n* = 9).

Five themes were identified including: *(1) The oppression of the illness; (2) Supporting the trial experience; (3) Making the most of now, ideally; (4) Hope is an antidote to desperation; and (5) The primacy of relationships.* The first theme contextualizes the experience of the caregiver. The second, third, and fourth themes are in response to this context, and the fifth theme is a response to the issues presented by the previous themes.

***Theme 1. The oppression of the illness*** sets the context for this work by capturing the breadth and depth of the impact of advanced cancer on the life of the caregiver. Participants reported multiple impositions on their lives. Usually, the shock of a diagnosis of advanced cancer marked the first significant emotional impact and, for some, the shock did not diminish over time. Ongoing impositions included the time and energy required to attend medical appointments and care for the patient in practical ways. Participants also reported how organising broader family demands can also be required and create a greater load because of the fragility of the patient. Managing communications to, and visits by, well-wishers and external agencies also required a lot from caregivers. For some, especially those with children, the loss of the patient’s salary meant increased financial pressure and the requirement to sometimes work longer hours. Such pressure was often compounded when financial decisions around medications required weighing up cost and potential benefit of treatment. There were also psychological demands such as supporting their close associate through existential anxiety and, for themselves, their own grief about the uncertain but expected death of their close associate. These pressures could lead to feelings of isolation for the caregiver. See Table 1 for representative quotes.

**Table 1.** Participant Quotes Supporting Theme 1

**The shock of the diagnosis:** “I just saw his demeanour and the look on his face when the surgeon said ‘I’m sorry.’ It was the shock. I’ll never unsee that face. It was horrific. Really.” (F, 40–60)
**Life revolving around the disease and extensive medical procedures:** “It just keeps going and going and going, all the kinds of treatments, the impact of chemo, which was devastating. All the chaotic health related stuff that came from chemo. The loss of mobility in all sorts of ways because of the peripheral neuropathy. And then she seemed to stabilise at that stage, and she had good results from the treatment. Then we noticed she wasn’t feeling great. And then eventually we found the cancer had come back. So she’s had, you know, the usual thing about chemo, surgery, radiation. And then when it returned, they found stuff in her spine and her hip and all sorts of places had flared up. And so she’s now every three weeks, forever it seems, been doing immunotherapy.” (F, > 60)
**Imposition on time and family life:** “That means that I’m just always on the go. Because he’s got the treatments and it’s Wednesday, 7:30, which means I get up about 6 and then get sorted and then drive to the North Shore, and then he’ll have the pump. So that means I’ll be around, and more and more conscious of how he’s doing. And he’s got his [elective therapy] once a week. And so on Friday, on the treatment week, I need to take him back to [medical centre] to have the pump removed. So that’s quite a bit with everything. And then also I’ve got my kids.” (F, < 40)
**Communications to friends and family:** “I did a huge amount of logistical organising and had a massive amount of communicating to people, like endless lists of people, and updating everyone and the managerials of it was vast.” (F, > 60)
**Financial pressure:** “I have my fulltime job and I [work] at night, so I am out several nights a week, because everything is so expensive, like for [winter sport] it’s $350 each kid.” (M, 40–60).
**Hard decisions about prioritizing finances:** “There’s a lot of financial pressure because the next treatment is one they won’t fund, so that’s $140,000 which gives [the patient] six months. So, we could remortgage, but then what? With young children, that’s really hard.” (F, 40–60)
**Living with uncertainty:** “This is the uncertain future. It doesn’t matter what aspect of life you’re talking about, the future is uncertain. When you have a terminal diagnosis, there isn’t a clear-up.” (F, > 60).
**Pervasive grief:** “I remember I was crying all the time and thinking, Ooooh, she’s going to die now and I am going to be by myself with the kids.” (M, 40–60).
**Revision of values:** “I stopped going to work. What life should be or the formula of life, none of that applies to us now. The entire way that I grew up and learned how to live life and try and be successful in life. There’s so many aspects just get thrown out the window.” (M, < 40)
**Psychological demands:** “And he said to me, ‘I think I’m dying but maybe I’m wrong,’ and I said, ‘Darling, I think you are dying.’ It felt really hard and then we both sobbed and it’s really, really hard. In between all that, there’s me having to watch my children’s emotions, which is really so hard for me.” (F, 40–60)

Importantly, as much as patient needs and wellbeing influenced the caregiver, so too did caregiver needs and wellbeing influence the patient, thus demonstrating their mutual vulnerability.

***Theme 2. Supporting the trial experience*** channeled the caregiver’s experience of watching the patient go through a trial investigating psychedelic-assisted MCP. This theme reflected numerous aspects of the trial progression, including decision-making in enrolling in the PAM trial, supporting someone whilst on the trial, and what they hoped would be lasting positive effects of the trial. Caregivers described being supportive in the decision to participate in the trial, with some encouraging participation with the expectation it would increase the patient’s feeling of being useful. Others admitted hesitancy about patients participating in a trial that used a psychedelic substance and first time therapy experiences. Caregivers also described watching their close associate find a broader sense of purpose in helping others by participating in the trial. After witnessing the patient show positive changes through MCP, including enhanced communication, caregivers expressed hope that the trial would enhance their dyad’s overall happiness. Lastly, caregivers highlighted the impact the experience of the trial had on them personally, and described challenges associated with having a close associate participate, such as carrying out the tasks required of them and disruption to usual life. See Table 2 for representative quotes.

**Table 2.** Participant quotes supporting Theme 2

**Supporting the decision to participate:** “My feelings wouldn’t have changed. You know, anything that she would feel would be helpful, I would go along with her.” (M, 40–60) “Oh, fully supportive because I want him to, you know, he’s never had therapy… I think it would be useful for him, it would be really good for him to find some meaning and less fear and a bit more peace about everything, yeah.” (F, 40–60) “I want her to spend time doing something that, you know she wants to do that as she gets enrichment and or enjoyment from, you know.. So she just wants to be doing something meaningful.” (M, < 40)
**Hesitancy at first:** “I was a bit nervous about it because it’s a powerful drug. It changes the way your mind, your brain, functions chemically, so I was a bit nervous about what would happen, and how that would affect her.” (M, 40–60) “My husband’s always a bit like cautious. Not so keen, but he’s always like, oh, if you think it’s helpful, then I’m going to try it. And I will say he’s really up for it, but I think he’s up for it because I just feel like you kind of need to have that process, so that you can move on.” (F, < 40) “I mean if there’s any part of this project that I have any concerns about it would be how he reacts to the counselling, not so much to the microdosing,’ cause that will be what it will be” (F, 40–60)
**Making a contribution to society:** “And I’m so glad that he took part in it. Feel like you know it’s making a contribution in a way. I just feel like it’s going to be beneficial to so many people. And I just think that’s kind of the right thing to do.” (F, < 40) “I think [the patient] felt good for having done something for the community. You know it was her way to give back into what is currently very high affect for her.” (F, 40–60)
**Excitement:** “I’m excited about talking about research…looking at quite a lot of things about how your mind is really influential and how the way you look at yourself and how you interpret and restrain your experience and how your state of being, so I was actually quite excited.” (F, < 40)
**The impact on the caregiver:** “Probably one of the harder things to do is talk about something positive in such a negative situation, and so you know to the credit of the trial, that opened up conversations for not only [the patient], but also, you know, for myself and her loved ones which, you know, predominantly the tough conversations when you have them.” (M, < 40) “She had her book and I think she could look forward, which was good. Which then helps me. If she knows and she has a plan then I don’t get the constant round of questions. It helps her, which then therefore helps me. So yeah, I think overall it was good.” (F, 40–60)
**Challenges in participation:** “So I suppose I found it made life a bit busier from my point of view as carer and partner because it was a Friday so it often felt like oh we can’t do something today, but that’s the case, we knew it was just a period of time.” (F, 40–60)

***Theme 3.*** In the context of the challenges noted in the theme 1, caregivers reported a desire to ***make the most of now… ideally.*** This theme highlights the expressed desire to do whatever might improve the quality of life for the patient in the time remaining to them. The oppression of the illness led to changes in perspective such that making the most of the present moment became a priority that helped them both manage the enormity of the advanced diagnosis. This meant acknowledging good things that happened and trying to arrange activities that would be enjoyable for them both. Keeping a wider perspective and not arguing about matters that are not important was commonly expressed as part of a desire to prioritise relationships and ensure that the time left was high quality. Caregivers also reported how taking part in a novel research trial was a way for them both to make the most of their time and to find meaning in paying it forward to people in the future who might find themselves in similar situations. They wanted to “*try to live the best life you can now*” (M, 40–60; see Table 3 for representative quotes).

**Table 3.** Participant quotes supporting Theme 3

**Change in perspective:** “I have to enjoy her as much as I can and enjoy the family. Like this week, we were playing cards in the room, like four or five hours just playing a game of cards. And this is very nice, just having everybody here. This is just full of joy, a little thing that we’ve started, that before the problem…. [thoughtful pause], yeah, we can play cards tomorrow or the next day.” (M, 40–60)
**Gratitude:** “It just becomes trying to get your mind in a position where you can acknowledge when the positive things are great and you can be happy in that moment. I think it’s probably what I’ve noticed. We’re trying to get the most.. We’ve been very fortunate.” (M, < 40)
**Making the most of the moment:** “I thought, OK, this guy’s not good, but fuck, let’s have fun together. Let’s live on the beach, let’s drink the wine and do the fun things” (F, 40–60).
**Acknowledging the ever-present cloud of mortality**: “Even when you get good news, you still don’t feel immediately better because you’ve still got this looming thing around you. I think once someone tells you that your partner or you are terminal, you have progress within that, but you can never get perfection because you know someone told you that, essentially, you’re going to die.” (M, < 40)
**Prioritising a good relationship:** “For me it just changed because all the little fiddly stuff that people argue or get upset over just doesn’t register anymore. You’re not shooting the shit over stupid things, right? You’re not wasting your time.” (M, < 40) “Small things like a miscommunication you’d argue about … have become so irrelevant … so you wonder why you even have those conversations, they’re so meaningless.” (M, 40–60)

The last 2 quotes in Table 3 indicate the conscious change in thinking by the caregiver, which inspired them to change their response as a means of improving the patient’s existential peace.

***Theme 4. Hope is an antidote to desperation*** describes how hope can give caregivers a degree of optimism that can counter feelings of desperation. Caregivers explained how hope helped keep them from feeling overwhelmed by the demands of the cancer and its treatments and by the misery of uncertainty and grief. Caregivers described how taking part in a novel research trial was a means of accessing hope either through the patient’s participation in the trial or their own. Such hopes were frequently associated with better health and good relationships. Often, sentiments were aspirational in hoping the trial would continue and would benefit others in the future. There was also the hope that they seemed to barely want to admit, that participation in the trial might somehow provide a miracle cure for their cancer. See Table 4 for representative quotes.

**Table 4.** Participant quotes supporting Theme 4

**Hope for the patient’s wellbeing:** “If she can feel just one ounce better, happier, more content, more accepting, that would be enough for me.” (M, 40–60).
**Hope for a miracle:** “We’re expecting a miracle. No, just joking.” (F, > 60)
**Hope for improved relationship**: “… and we can find a better way to approach the whole thing as a couple and so I can help .. And we both have to get help through it. You know, it’s not just his, it’s sort of a joint thing.” (F, > 60)
**Hope that the effects of the PAM trial will endure:** “I hope the kind of benefits that we’ve all taken from it, I hope that lasts. I think they probably will. Yeah, I think I can’t see us unlearning what we’ve learned.” (F, 40–60) “It just confirmed that [the death of the patient] was going to happen, and this trial was interesting in terms of actually offering some ways of coping with it, not just drug-related, but also just change in attitude.” (M, > 60)

***Theme 5. The primacy of relationships*** conveyed the precedence given to good relationships by caregivers after the diagnosis of advanced cancer. Caregivers observed that the terminal nature of the illness changed their relationship with the patient in several ways, sharpening its importance. The patient’s need for connection intensified, impacting the caregiver’s feelings towards the patient, and leading to the caregiver finding meaning in the relationship with the patient. One insight that changed the behavior of those who were caregivers to PAM participants was the realization that the relationship with the advanced cancer patient was of the utmost importance and that negative interactions were detrimental to the wellbeing of both. Largely, caregivers reported this realization followed the patient’s diagnosis. Patients seemed to report this realization after participation in the trial. It became apparent that the experience of participating in the trial heightened most patients’ awareness of the salience of good relationships in terms of their existential wellbeing. See Table 5 for representative quotes.

**Table 5.** Participant quotes supporting Theme 5

**The poignancy of the relationship post-diagnosis:** “it obviously changed our life, but it changed our relationship as well, in terms of just that commitment to each other” (M, < 40).
**Prioritising relationships:** “I think he started to think more about what kind of life he wants and how he can actually be more present for his family and especially for our children. Before, he was always really focused on working and always wanted the time for himself. After whole days working you find the children annoying. You want them to get away from you, but he’s more conscious and he wanted to spend more time.” (F, < 40)
**The influence of the trial on relationships and existential peace:** “I think he has a better perspective. He’s really taken it to heart, the lessons of the therapy about bringing his best self to the situation, facing it with courage and love for his family. That’s noticeable. How much he kind of cherishes us all.” (F, > 60) “He is a bit better at listening and standing back a little bit … He’s calm, he’s peaceful, he doesn’t get as upset as often. He’s a bit more well-rounded … and thinking of us .. He’s able to be present and support me.” (F, 40–60) “I think we are better. We are facing up to it with more honesty for start. We can talk about things that we couldn’t go near before, acknowledging that his life is going to be shorter and all that stuff.” (F, > 60) “We’ve got used to actually now just stopping and listening more carefully… so that we don’t end up in arguments all the time which has been going for 30 years. So, we’re sort of, yeah, calming down a wee bit.” (M, > 60)
**The benefit of good relationships for existential peace**: “Now she’s definitely more conscious of making sure things are meaningful in the time she spends with her loved ones, even just letting things go. Things that you would have spent half an hour arguing on, you don’t spend time doing it. We all argue about stupid shit. But I think for her, probably one thing that stands out to me, is her interactions with the people that she loves. There’s way more time now just focused on positive, healthy, loving interactions, which I think is how it should be and so it’s really helped guide her towards a bit of that.” (M, < 40) “So did it help him? I think he’s happy. I think he’s pleased he’s done it and I think it probably has helped him frame life, the universe and everything, just better. So in terms of what’s the most important thing, it’s his son and me, it’s staying alive as long as he can for us.” (F, 40–60)

Through their involvement in the PAM trial, caregivers reported how they had reshaped their responses with the person they were supporting and had generally attempted to reject interactions that were not constructive.

## Discussion

The current research is the first to explore caregiver experiences of supporting a patient undergoing a trial investigating LSD microdosing alongside MCP and has implications more broadly. Our findings highlight the bidirectional relationship between caregiver and patient, where influence on one becomes an influence on both. This research reinforces that caregiving pervades almost every aspect of the caregiver’s life, with their wellbeing closely aligned with that of the patient. We found that caregivers were generally supportive of their close associate participating in the trial, with some highlighting the impact it had on them personally. Caregivers described a desire to make the most of now and referred to the role of MCP as a means of accessing hope, improving the dyad relationship, and reducing existential distress. This study extends the literature by demonstrating that the caregiver experience is influenced by the patient experience of placebo or psychedelic-assisted talk therapy, reaffirming the importance of holistic cancer care.

The present findings confirm the bidirectional impacts on patient and caregiver wellbeing that has been shown in previous research. Such impacts include emotional strain, practical load, financial pressure, and psychological demands. Similar research has shown the significant physical and psychological impact an advanced cancer diagnosis has, not only on the patient, but also on their informal caregiver (Williams 2014; Pedreira et al. 2019). Likewise, the caregiver also impacts patient experience (Augeraud-Véron and Leandri 2024). In the context of the PAM trial, the relationship between caregiver and patient played a significant role in the outcomes for both because of their close partnership and mutual influence.

Caregivers in the present study described varying degrees of influence in the decision to participate in a trial investigating psychedelic-assisted MCP, with some offering encouragement and others admitting hesitancy about microdosing due to concern related to psychedelics. Despite an escalating interest in the potential for psychedelics to be of benefit in various clinical contexts, stigma, and misconceptions remain (Viña 2024). So while this finding is unsurprising, it has important implications for clinical trials administering psychedelic drugs. Caregivers influence patient decisions and if fearful of what participation might mean, may dissuade their close associates from taking part. Research has found that greater awareness of psychedelic drugs by cancer patients (Best et al. 2024), healthcare clinicians (Nuha et al. 2025), and cancer clinicians (Reynolds et al. 2024) increases openness to the idea of their therapeutic potential. Education about psychedelic drugs has the potential to support informed decision-making that is based on fact rather than misconceptions.

In the current work, caregivers described how the patient’s participation gave both patient and caregiver the opportunity to make altruistic contributions to cancer research. This finding fits with other research that shows altruism is a primary motivator for participation in cancer clinical trials (Nielsen and Berthelsen 2019), and that participation in research can increase altruistic behavior (Williams et al. 2008). Increases in altruism can provide a sense of meaning, purpose, and greater pro-social behavior for patients and caregivers (Hawley 2014), particularly in patients with poor cancer prognoses (Nielsen and Berthelsen 2019). The talk therapy utilized in the PAM trial (MCP) has been shown to be effective in reducing psychological distress, increasing meaning, and improving social relationships in cancer populations (Vos and Vitali 2018; Shen et al. 2025). In line with previous research (Pedreira et al. 2019), caregivers reported greater wellbeing when their close associate experienced positive outcomes of the trial. Thus, participation in the PAM trial appeared to provide an avenue to increase meaning and channel altruistic feelings for both patient and caregiver.

Caregivers in this study also described a desire to make the most of now, doing whatever might improve the quality of life for the patient in their remaining time. Many were explicit in articulating the idea of not wasting time on negative interactions as a crucial means of improving relationships and quality of life for the patient. Other research suggests that increasing openness and communication is likely to enhance relationships with those most closely associated with the advanced cancer patient (Otto et al. 2021). Because psychedelic therapies can enhance empathy, relatedness to others, and meaning-making which is associated with greater wellbeing (Cavarra et al. 2022; McManus et al. 2022), microdoses of LSD may be a means to that end. It is possible, too, that the benefit may be broader than the patient and also benefit the caregiver through greater empathy and communication, enhancing quality of life for both members of the dyad.

Caregivers described how hope can counteract feelings of demoralisation, and many saw taking part in the PAM trial as a means of accessing hope. Demoralization, characterized as hopelessness and helplessness due to loss of meaning in life, is a common presentation in cancer patients (Bobevski et al. 2022), with 13–18% of cancer patients experiencing clinically significant demoralization (Robinson et al. 2015). Similar to other research showing that participation in clinical trials can provide hope in patients (Godskesen et al. 2015), taking part in the PAM trial appeared to also offer hope for caregivers. As the PAM trial was a feasibility trial, it was not powered to detect statistically significant changes in demoralisation, however, caregivers reported that participating in the trial provided them means to access hope.

Most striking was the universality of caregivers’ reports that there was no time to waste with negative communications within the dyad. Knowing that life may be time-limited can cause shifts in perspectives, consequently making patient–caregiver dyads less focused on small negatives (Otto et al. 2021). Caregivers reported consciously altering their communications in positive ways, which strengthened their relationships and contributed to a greater sense of existential peace in the patient. However, it is also important to note that the pressure of valuing every detail and not addressing disagreements can cultivate unrealistic pressures within the relationship (Thomson et al. 2024). Cancer is inevitably stressful, and stress inherently brings about disagreements (Siminoff et al. 2012). While open communication has been shown to improve caregiver–patient congruence (Li and Loke 2014), expectations around maintaining positive communications should remain grounded and achievable for cancer dyads.

Caregivers in the current study reported that taking part in the clinical trial improved their relationships which benefited both caregiver and patient. In some cases that was achieved through greater honesty and willingness to discuss difficult topics after the patient had participated in the psychotherapy. Previous research has recommended that relationship quality be included in investigations of end-of-life care as it can predict caregiver burden (Reblin et al. 2016) and help understand how cancer communication can benefit both caregiver and patient (Otto et al. 2021). The current qualitative work provides further evidence that enhancing relationships through trial participation has potential to improve outcomes for both the caregiver and patient.

### Clinical implications

This research confirms the significant emotional, social, physical, and practical stress that cancer patients and their caregivers face. However, the limited effectiveness and overall lack of interventions addressing existential distress among patients is a significant unmet need in palliative care (Ross et al. 2022). Attending to existential distress in cancer patients at the later stages of life should be a priority in improving quality of life in this population (Breitbart 2002; Ross et al. 2022). The current research evidenced that caregivers perceived positive changes in patients participating in a trial investigating LSD-microdosing alongside MCP, suggesting that it might be a useful approach in reducing distress for patients. The current qualitative research also suggests support for trial participation in improving the wellbeing of caregivers. Future clinical trials should ensure caregivers are included alongside patients so that the breadth and depth of the cancer experience is captured.

Pertinent to the current work is research showing that psychedelic-assisted or placebo-assisted psychotherapy might mitigate existential distress as well as improve relationships. It had been suggested that psychedelic-assisted psychotherapy has the potential to target biopsychosocial-spiritual dimensions of caregiver distress (Gold et al. 2023). While most cancer care focuses on patient wellbeing, caregiver wellbeing should also be prioritized, not least because their care duties are underrecognized and undervalued as essential work (Williams 2014). In combination with the personal benefit to the caregiver, improved caregiver wellbeing is also beneficial for the dyad: a stressed caregiver is less able to provide effective assistance to the cancer patient and stress-laden caregiving is likely to have deleterious effects on the physical and psychological health of the caregiver (Pedreira et al. 2019). Delivering MCP to caregivers has already shown benefit (Applebaum et al. 2022), and it is important to consider the potential benefits in offering novel psychological interventions to both patient and caregiver because of their mutual vulnerability. Further clinical trials are needed that investigate psychedelic-assisted interventions that support caregivers as well as the patient.

## Conclusion

The potency of the patient–caregiver relationship means that the benefits of interventions supporting patients will be enhanced by also including the caregiver. In this study, caregivers described the value of MCP in helping patients to talk honestly and openly about matters relating to death that were previously too uncomfortable to broach. Reports from caregivers suggested that participation in a trial investigating MCP alongside LSD microdosing seemed to help address existential distress in advanced cancer patients although noting that the qualitative design of the current study precludes definitive conclusions on this. Further research could include similar interventions specifically for the caregivers themselves. In holistically caring for cancer patients, the caregiver, and the wider family should have access to hope, support, and relationship-enhancing interventions.

## Data Availability

The dataset used and analyzed during the current study is available from the corresponding author on reasonable request.
